# Design and Analysis of a New Multi-Part Composite Frangible Cover

**DOI:** 10.3390/polym15153307

**Published:** 2023-08-04

**Authors:** Yuan Qian, Wenlong Li, Xiaopei Wang, Deng’an Cai

**Affiliations:** 1 Purple Mountain Observatory, Chinese Academy of Sciences, Nanjing 210034, China; 2 State Key Laboratory of Mechanics and Control for Aerospace Structures, Nanjing University of Aeronautics and Astronautics, Nanjing 210016, China; 3 Chinese Flight Test Establishment, Xi’an 710089, China; 4 Nanjing Research Institute on Simulation Technique, Nanjing 210016, China

**Keywords:** composite material, multi-part frangible cover, structure design, separation process, finite element analysis

## Abstract

In this paper, a new multi-part composite frangible cover (MCFC) was designed and fabricated. The frangible cover, manufactured with a traditional manual lay-up method, is designed to conduct a simulated missile launch test using a specially developed test device. A weak zone structure of the composite multi-part frangible cover was designed, and the separation process of the cover was studied by numerical simulation. Based on the strength envelope of the weak zone and the equal-strength design principle, a design method for the weak zone structure of the composite multi-part frangible cover was proposed. A finite element model of the composite multi-part frangible cover was established, and the separation process was numerically simulated and analyzed. Afterward, the verification experiments were carried out. Close agreements between the numerical and experimental results are observed.

## 1. Introduction

As an important part of a missile storage and launch system, a missile-launch canister cover is a storage device for an inner missile to protect a warhead from damage and prevent a leak of inert gas before the missile is launched. The cover should be opened promptly during the missile launch process, not affecting a normal launch. Since the early seventies, many attempts have been made by various researchers to develop different metal missile-launch canister covers, which were opened mechanically or burst [1,2,3,4]. Major drawbacks of these metal canister covers are the complexity of their mechanism, difficulty in manufacturing, ponderous structure, high cost, etc., which can directly affect the stability of the covers. Therefore, there is a need to seek and study more reliable alternatives to these covers.

The launch container acts as a pressurized vessel or pipeline when the missile is stored and transported. A series of studies on the performance of pressure vessels and pipelines have shown that the type and location of defects have a non-negligible effect on their pressure-bearing performance. Alizadeh et al. [5] reported that greater crack width caused lower critical pressures and that an external crack had more influence on the vessel’s capacity than an internal crack of the same configuration. Zhang et al. [6] found that the failure pressure of a pipeline depended on both the longitudinal spacing between internal and external defects and the ratio of the internal defect depth to pipe wall thickness when both external and internal defects were present. Usually, the special opening load and opening mode of frangible covers are realized by creating weak areas in their relative locations. The influence of defects on the load-bearing performance of pressure vessels provides an idea for the fabrication of weak areas of frangible covers.

Composite materials are replacing metals in aerospace applications due to good properties such as a high strength–weight ratio, good corrosion resistance, and long fatigue lives [7,8,9,10,11,12,13,14,15]. Many canister covers for missile launch systems are gradually being manufactured with composite materials. In order to improve reliability in combat conditions, shorten the reaction time, and increase the firing speed, scholars have conducted some research on the structural design and mechanical properties of the composite frangible cover. Design and manufacture of frangible covers using fiber-reinforced composites have gradually become mainstream research directions. Doane [16] designed a film cover using glass fiber-reinforced epoxy resin composites whose opening method was penetration. Its weak areas were fabricated by pre-made scoring. The film cover could tear along the scores under impact by the warhead during missile launching. However, this film-type cover had poor resistance to deformation under pressure load due to its small stiffness. Wu et al. [17] and Kam et al. [18] developed a frangible laminated composite canister cover that would not cause any damage to the missile during its emission process. The cover would burst open in accordance with a predetermined pattern, allowing the missile to fly out of the canister unharmed when subjected to an internal impulsive pressure generated by the missile engine. A phenomenological Tsai–Wu failure criterion was used theoretically for strength prediction of the canister cover with a static finite-element (FE) model. The accuracy of the Tsai–Wu criterion in predicting first-ply failure of laminated composite plates was verified in their previous studies [19,20,21,22]. Zeng et al. [23] studied the failure of a fly-through frangible cover consisting of five plan-liked parts via transient dynamics finite element analysis. Numerical results showed that the failure pressure and time rose as the length of the bonding layer increased. Cao et al. [24] and Cai et al. [25] designed a round cap-shaped composite frangible cover whose microstructure in the weak area was a combination of bonding and lap joints. A parametric design method for this weak-area structure was proposed based on numerical analysis and experiments. The deflection and failure pressure of this type of frangible cover were investigated. Zhou et al. [26] proposed a frangible composite canister cover with the function of specified direction separation whose weak zone structure had a local non-split area. Cai et al. [27] established a transient-dynamics model based on the approximate Riemann algorithm for the failure analysis of a frangible composite canister cover. They analyzed the influence of the positions of the weak zones and the length of the bonding layers on the failure pressure. Wang et al. [28] designed a penetrating glass-fiber frangible cover with scratches. They used the finite element method to simulate the process of a rocket breaking through a frangible cover and gas flow impinging on the frangible cover. Cai et al. [29] investigated the abnormal failure of epoxy foam frangible covers under external pressure using finite element simulations and experiments. The results indicated that buckling instability failure was the main reason. Xu et al. [30] designed a new circular plate frangible composite cover that had a weak zone with an embedded bonding structure to achieve the required one-way locking function. This cover could achieve a high ratio of external to internal load-bearing limits under the premise of a compact structure and small occupation.

Furthermore, the biggest advantage of composite materials is their designability. Not only will the material of the fiber and matrix affect the performance of composite materials, but also the difference in fiber content and the change in lay-up sequence will greatly affect the performance of composite materials. Therefore, according to the load conditions and structure form, by choosing the appropriate optimization algorithm to reasonably select the lay-up angle, lay-up thickness, and lay-up sequence, the lightest structure can be achieved under the requirements of product performance. Lin et al. [31] applied an improved genetic algorithm in the field of structural optimization of composite laminates to the lay-up sequence optimization of sandwich plates and composite propellers. The results showed that the time consumption of the improved genetic algorithm was reduced by about half. Kang et al. [32] used a modified genetic algorithm to find the optimal solution of the structure when studying the post-buckling strength of constrained composite stiffened plates, taking the lay-up angle and lay-up thickness as the design variables and weight and post-buckling strength as the objective functions. Abouhamze et al. [33] established a multi-objective optimization system for the natural frequency and buckling load of composite cylindrical plate structures. A genetic algorithm and neural network were used to obtain the layering thickness under preset angles. Then a genetic algorithm was used to optimize the layering sequence under such circumstances. Omkar et al. [34] adopted the cloning algorithm in the artificial immune system to carry out multi-objective optimization of composite laminates under different loads (unidirectional, biaxial, and bending loads). With lay-up thickness, lay-up sequence, and single-layer thickness as design variables, the lightest weight and the minimum number of composite components were achieved on the premise of satisfying strength. The design variables of composite structure optimization include layering angle, layering thickness, and layering sequence. Compared with other isotropic materials, the structural optimization design variables of composite materials have obviously changed, which also reflects the characteristics of composite materials. Therefore, a new type of brittle composite cover can be designed according to composite structure optimization design characteristics.

It is considered that there is a problem with the large separation weight of the broken composite cover, which needs to reduce the weight of the thrown part of the cover. In this paper, a new type of multi-part composite frangible cover (MCFC) is designed to meet these strictly new requirements. Experimental research and numerical analysis are then carried out. The influence of key structural parameters on the mechanical properties of the multi-part cover is also discussed. Additionally, experimental studies of the failure pressure of the MCFC were performed in the simulated launch setup to validate the numerical results.

## 2. Structure Design

### 2.1. Main Structure

**Figure 1:**
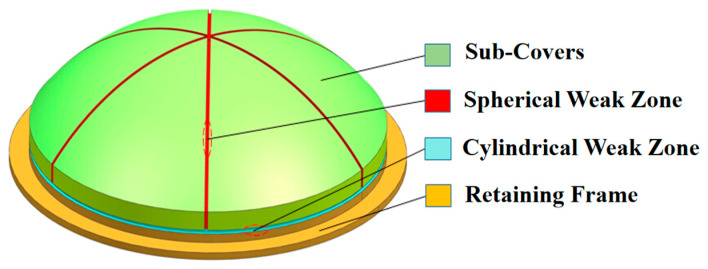
Structure of the multi-part composite frangible cover.

**Figure 2:**
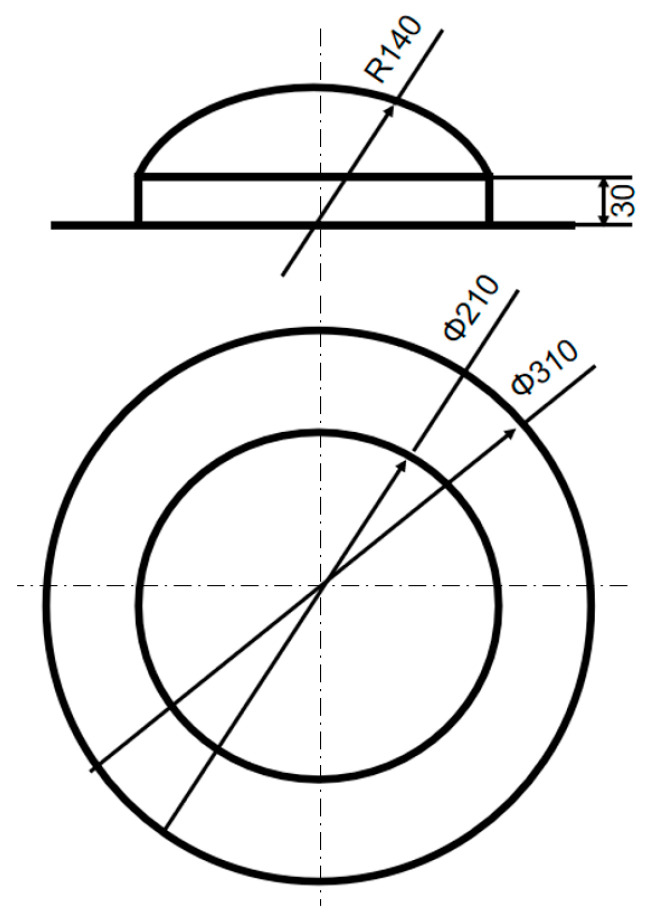
Dimensions of the multi-part composite frangible cover.

The MCFC was designed as a hemispherical structure, as shown in Figure 1 and Figure 2. The weak zones divided the frangible cover into seven pieces. Fiberglass cloth combined the pieces together in the weak zones. The MCFC mainly included four functional parts: a retaining frame, a spherical weak zone, a cylindrical weak zone, and six sub-covers. The frangible cover was fixed to the launch canister through a retaining frame to play a sealing role during missile storage. When the missile is launched (after ignition), the gas flow generated will cause the air pressure in the launch canister to rise. The weak zone of the frangible cover was broken due to high pressure, and then six sub-covers flew away.

### 2.2. Structure of Weak Zones

**Figure 3:**
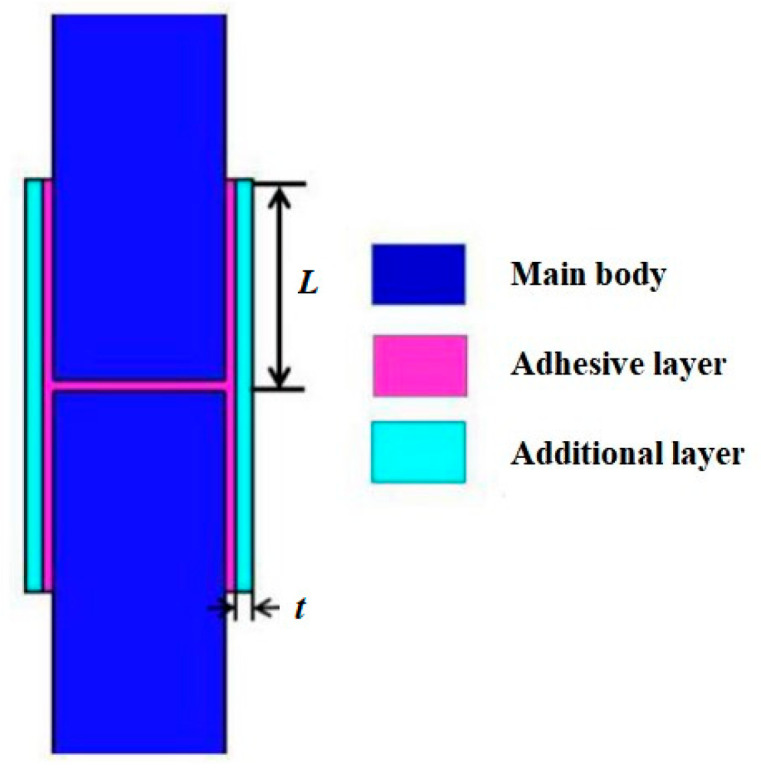
Structure of the weak zone.

The spherical weak zone and the cylindrical weak zone were both designed as composite double-lap structures, as shown in Figure 3. The strength of the weak zone was a key parameter that determined the performance indexes of the MCFC. The lap length and lap thickness between the additional layer and the main body were defined as *L* and *t*, respectively. *L* and *t* were the critical parameters that determined the strength of weak zones. In this paper, the structural strength design of the weak zone was realized by adjusting *L* and *t*. Unreasonable lap parameters could cause some weak zones to be damaged before the other parts. Asynchronous failure of the weak zones was easy to cause the air leakage of the launch canister and the failure of the sub-cover separation. Furthermore, the normal launch of the missile would be affected.

Based on the above reasons, the design principle of equal strength was proposed to ensure the bursting performance of the MCFC. The parameters were determined based on the local stress value (Von Mises stress in this paper and hereinafter inclusive). The frangible covers of different weak zones were destroyed simultaneously under bursting pressure.

**Figure 4:**
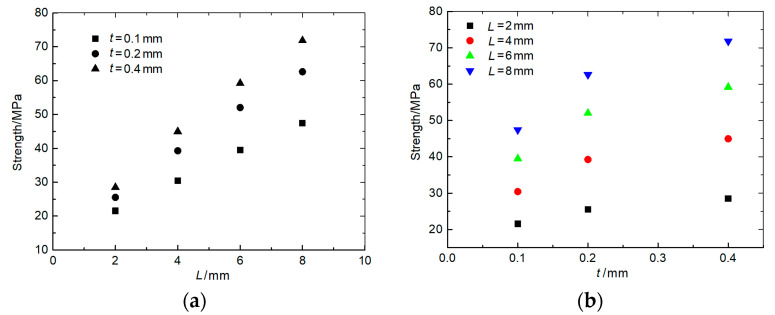
Tensile test results of the double-lap structure: (**a**) Effect of *L* on tensile strength; (**b**) Effect of *t* on tensile strength.

The variation trend of the strength of the weak zone with *L* and *t* was studied through experiments. The test results are shown in Figure 4. Figure 4a shows that when the lap thickness *t* was in the range of 0.1–0.4 mm, the tensile strength increased approximately linearly with the increase in the lap length. Figure 4b shows that when the lap length *L* was in the range of 2–8 mm, the tensile strength increased with the increase in lap thickness, but the growth rate gradually decreased.

**Figure 5:**
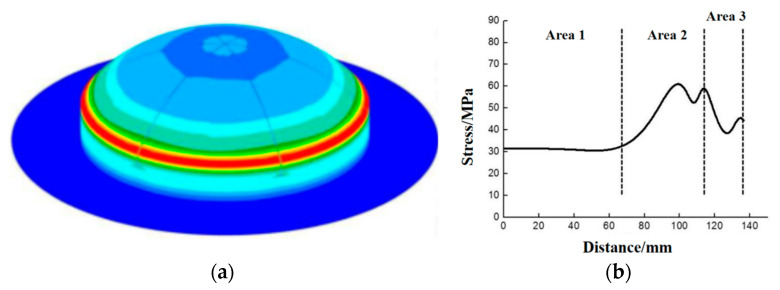
Stress distribution of the multi-part composite frangible cover: (**a**) Stress variation of the spherical weak zone from top to bottom of the cover; (**b**) Stress distribution in the cylindrical weak zone.

The stress distribution of MCFC acting under internal pressure was calculated by the finite element method. The calculated results are illustrated in Figure 5. In Figure 5a, the stress distribution in the cylindrical weak zone was uniform because the cylindrical weak zone and internal pressure were distributed symmetrically along the hemispherical cover. On the other hand, Figure 5b shows the stress variation of the spherical weak zone from top to bottom of the cover. The curve shows that the stress-changing scale is small in the upper-middle part of the spherical weak zone. After entering the corner zone, there is stress concentration in the middle and bottom of the spherical weak zone.

**Figure 6:**
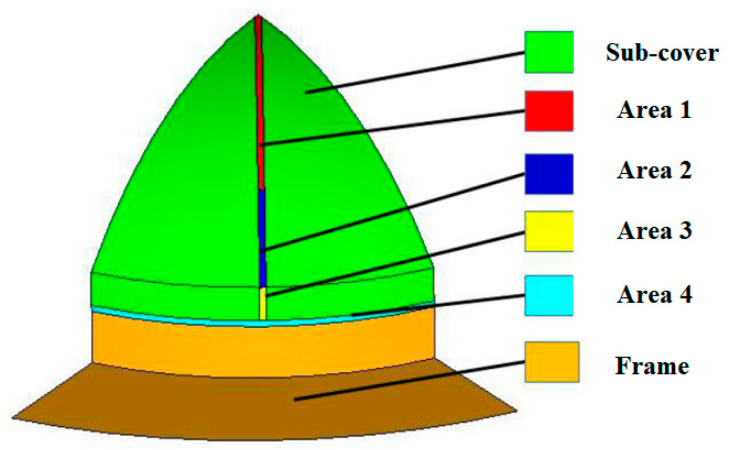
Region division of the weak zones.

According to different stress levels, the weak zones could be refined into four zones, as shown in Figure 6. Area 1 was a spherical, weak area away from the corner. Area 2 was a weak spherical area located on the spherical surface near the vertical edge. Area 3 was a spherical surface located on the vertical edge of the weak zone. Area 4 is a cylindrical weak zone. The regional division is shown in Figure 6.

Based on the relation between lap parameters and the weak zone strength, and taking into account the equal strength design criterion, the lap length *L* and lap thickness *t* of different areas are given in Table 1.

## 3. Numerical Analysis

### 3.1. Ply Optimization

The MCFC in this paper was prepared with glass fabrics as reinforcements and epoxy as matrix resin. An optimum design was obtained by calculating different thicknesses and different stacking sequences of the laminate using Isight software.

The ply thickness optimization of MCFC was a single objective optimization problem with certain constraints. The weight of MCFC was optimized in terms of different parameters of ply thickness. There were two constraints. One was that the maximum deformation of the frangible cover was less than 1 mm under the internal pressure of 0.15 MPa, and the other was that the stress in the two weak zones was less than 10 MPa.

The optimized ply thickness is listed in Table 2. The weight of the optimized MCFC was approximately 391 g, which was 28.1% lighter than that before optimization. The lightweight design of MCFC could dramatically reduce the threat to the safety of both machines and personnel.

The stress level of MCFC was optimized in terms of different parameters of ply angle. The constraint conditions were the same as for the ply thickness optimization. Also, the lay-up design considered the following factors: (1) In order to reduce internal stress and thermal warping, symmetrical laminates are often used in engineering; (2) the fiber direction of the ply should be consistent with the tensile and compression directions of the structure, making the best use of the strength and stiffness characteristics of the material fiber direction; (3) from the perspective of stability and impact resistance, a 45° ply should be used on the outer surface of the laminate; (4) the ply group in the same direction should not exceed four layers; and (5) the angle difference between adjacent layers should not exceed 60°.

The optimized ply angles and sequences are listed in Table 3. The maximum stress of optimized MCFC was approximately 33.4 MPa, which is 13.4% less than the primary structure. The ply angle design of MCFC could distribute the distress evenly. Furthermore, the stress and maximum deformation in the weak zone were reduced to a certain extent under the premise of meeting the constraints.

### 3.2. Separation Process of the MCFC

The radial dimension of MCFC was much greater than the thickness. Accordingly, orthotropic shell elements were used for the retaining frame and the sub-covers. The results of tensile tests of weak zones displayed that the fracture mechanism of a typical specimen was a brittle fracture without obvious plastic deformation.

The separation behavior was a transient process. The finite element model was simulated by the explicit kinematic analysis module of ABAQUS/Explicit. The key to the simulation analysis of the fragile cover separation process lay in the strength check of each weak zone and the treatment of the failed elements. The constitutive model of weak zones was compiled by the Vectorized User-Material (VUMAT) subroutine. Von Mises criterion was adopted to indicate whether failure happened or not. As the Von Mises’ equivalent stress approached the strength of the weak zones, the failure elements were deleted to simulate the separation behavior of sub-covers.

**Figure 7:**
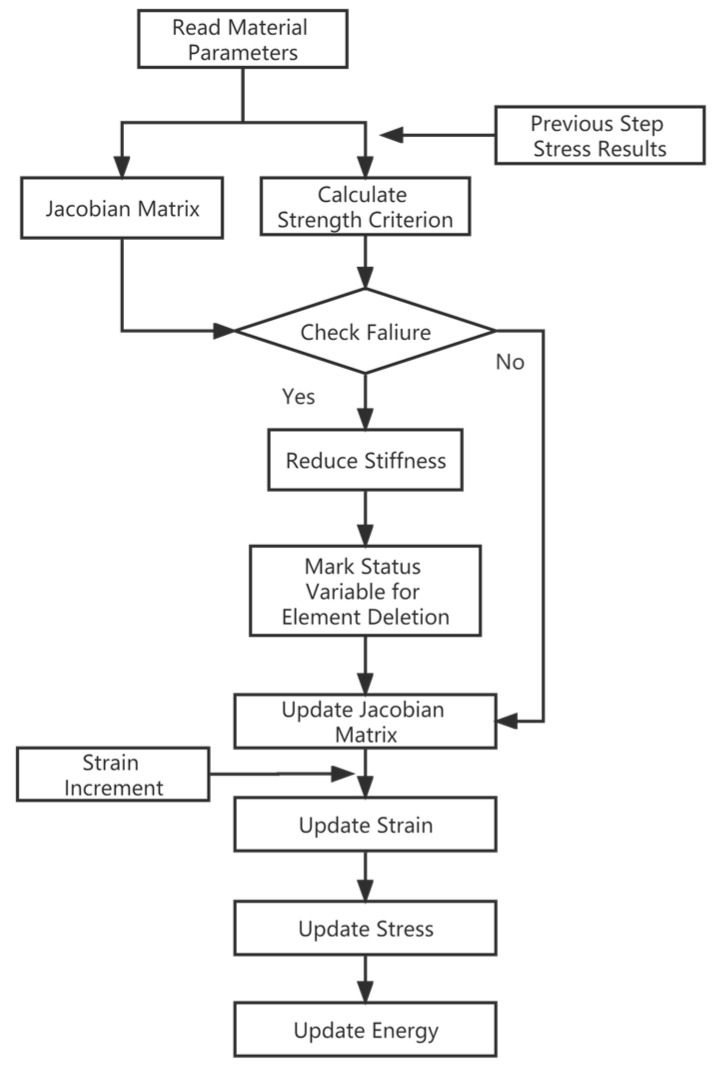
Flow chart of finite element analysis.

Due to the expensive costs and limited experimental data, the finite element method (FEM) is widely used to predict the load capacity and investigate the progressive damage and failure behavior of composites [35,36,37,38,39,40,41]. The finite element calculation process used to simulate the breaking process of the MCFC is shown in Figure 7. Firstly, a finite element model of the MCFC is established, the flange is fixedly supported, and the impact load changing with time is applied to the inner surface. An explicit solver is used to calculate the stress of the element. The failure criterion of VUMAT is written to determine whether the weak area unit fails. If a failure occurs, it will be deleted. With the advance of the ABAQUS incremental step, the dynamic process is simulated explicitly.

**Figure 8:**
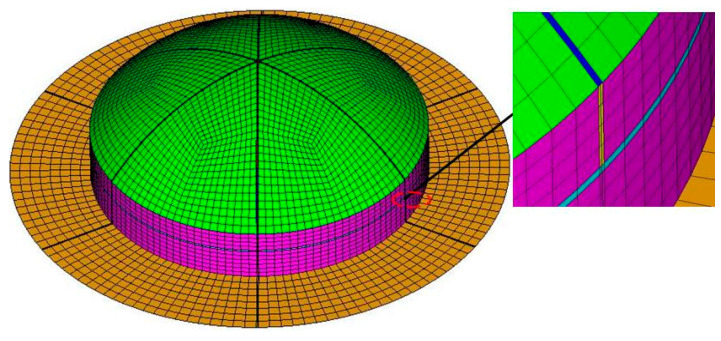
Finite element model of the multi-part composite frangible cover.

The strength of the main body of the MCFC was much greater than that of the weak zones. Therefore, the damage to the sub-covers and the retaining frame was not considered. The meshes of MCFC are shown in Figure 8. It was modeled with 5798 nodes and 5472 elements (S4R). The width of elements in the weak zones is 1 mm.

The clamped boundary condition was applied to the retaining frame to simulate the installation of MCFC. The frangible cover was broken under the pressure generated by the wake when the missile was launched. The impact load was equivalent to uniform pressure with respect to time. The uniform pressure changed over time in the form of a sawtooth wave. The peak load of the impact was 0.8 MPa. The acting time of the impact load was about 4 ms. The total calculation time and incremental step time were set to 5 ms and 0.1 ms, respectively.

Based on the test results of composites in the laboratory, the mechanical properties of the main body of MCFC are given in Table 4. These results were obtained by testing the composite plates prepared by the same manufacturing process. The elastic modulus of the weak zones was 3.5 GPa, the same as that of the epoxy resin. The Poisson’s ratio of the weak zones was 0.35. The strength of the weak zones varied depending on the lap parameters, which can be obtained from Figure 4.

**Figure 9:**
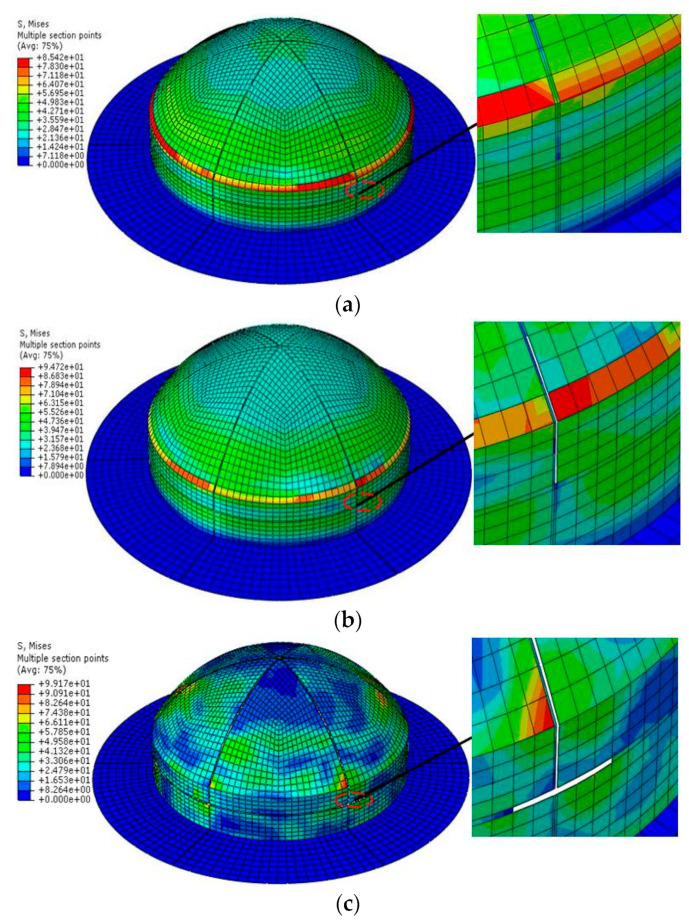

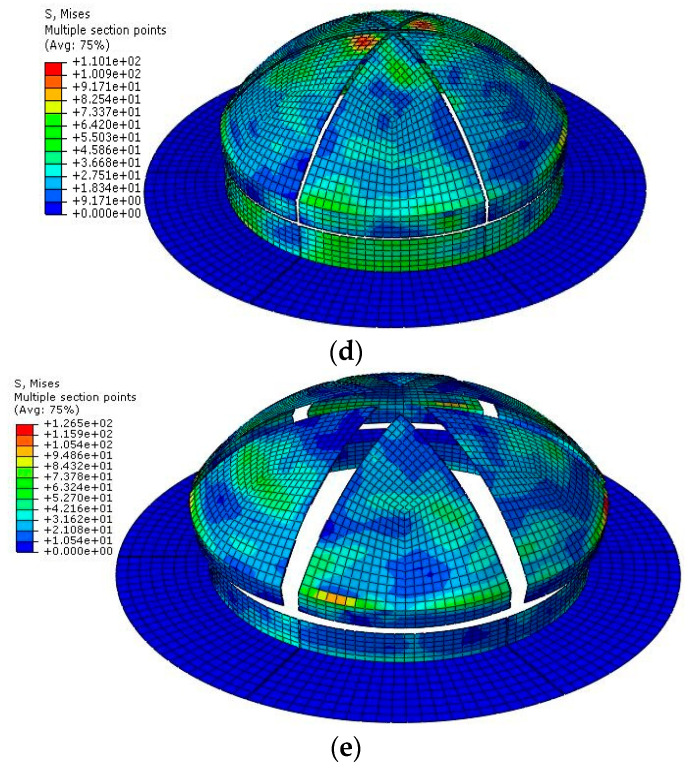
Failure process of the multi-part composite frangible cover: (**a**) *t* = 3.6 ms; (**b**) *t* = 3.7 ms; (**c**) *t* = 3.8 ms; (**d**) *t* = 3.9 ms; (**e**) *t* = 4.0 ms.

The separate process of MCFC is shown in Figure 9, as described below. (1) There was no failure in the structure of MCFC at 3.6 ms. However, there was a stress concentration in Area 3. (3) Failure occurred in Area 3 at 3.7 ms because of the stress concentration. And it showed an expanding trend from Area 3 to Area 2. (3) With further increasing pressure, the elements of the spherical weak zone were all deleted at 3.8 ms. Furthermore, the failure extended to the cylindrical weak zone. (4) All the weak zones had been destroyed at 3.9 ms. The separation happened between the sub-covers, the retaining frame, and the sub-covers. The sub-covers flew away under the pressure of 0.78 MPa. (5) It shows that the stress cloud diagram of the frangible cover after the weak zone was completely destroyed at 4.0 ms. Since the load did not disappear and the dynamic response was hysteretic, the overall stress still increased after the sub-covers were separated without constraints.

According to the above analysis, at 3.7 ms, when the load is 0.74 MPa, the weak zone at the corner of the frangible cover was the first to fail and expand to the periphery. At 3.9 ms, when the load was increased to 0.78 MPa, the weak zone was completely destroyed, and the sub-covers of the separated bodies flew out in all directions. It took only 0.2 ms for the weak area to go from damage to complete destruction of all the weak zones, and the frangible cover separated quickly, indicating that the structural design of these weak zones was reasonable. At the same time, compared with the design index of the breaking pressure of 0.8 MPa, the breaking pressure obtained by simulation is 0.78 MPa, which is very close to each other. It verified the feasibility and accuracy of the structure design scheme of MCFC in this paper. However, compared with the design value, the simulation result is a little small because the parameters of each weak zone are selected according to the stress distribution calculated by static analysis. Additionally, there is a certain stress concentration in the frangible cover, which leads to the early failure of this area.

## 4. Experimental Study

### 4.1. Manufacturing Process

**Figure 10:**
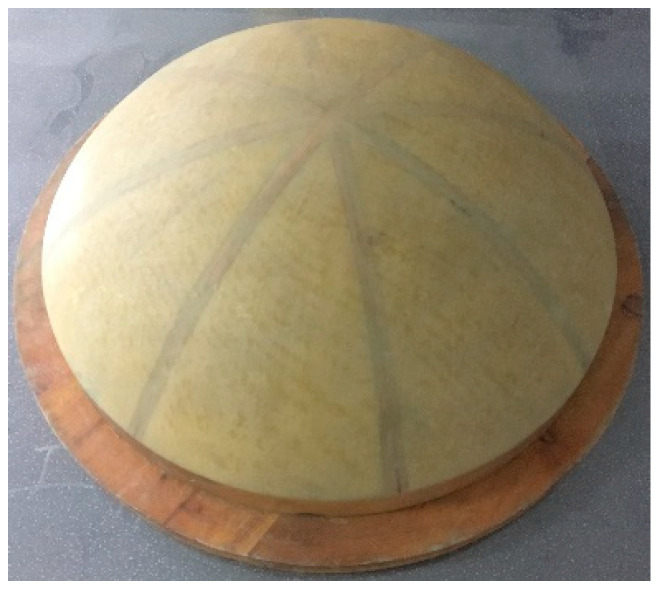
Mold of the multi-part composite frangible cover.

**Figure 11:**
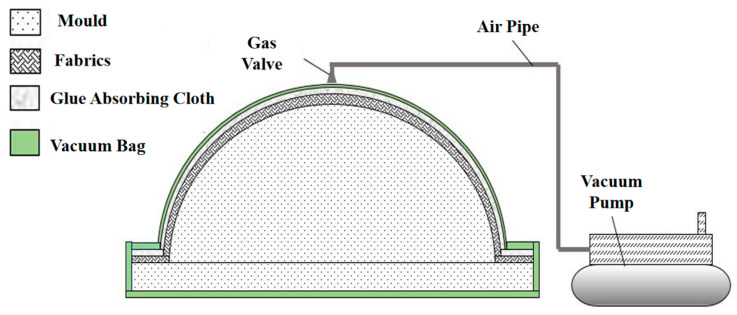
Vacuum-assisted resin infusion (VARI) of the multi-part composite frangible cover.

A convex mold was used to manufacture the MCFC to ensure dimensional precision and surface quality, as shown in Figure 10. The manual lay-up process was widely used in composite production to produce small batches and multiple products without limiting their size or shape. But the instability of the manual lay-up process could lead to the delamination of a composite laminate. For this reason, the vacuum-assisted resin infusion (VARI) method was introduced to aid the manufacture of MCFC. With the above factors, the manual lay-up process and the VARI method were combined to form MCFC. The diagram of VARI is shown in Figure 11.

**Figure 12:**
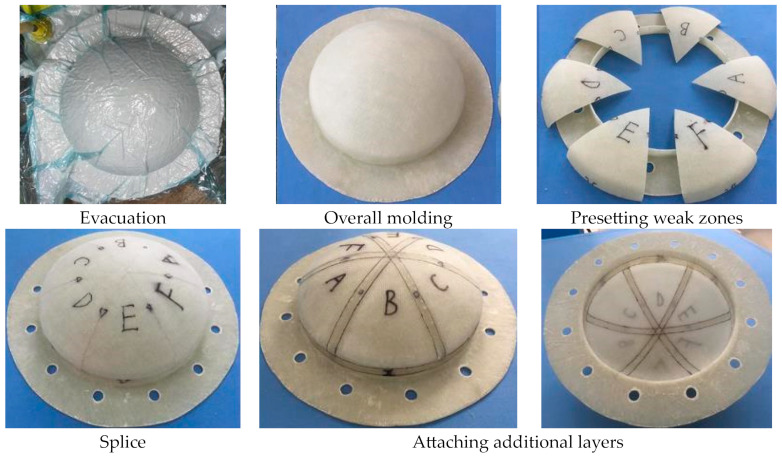
Manufacturing process of the multi-part composite frangible cover.

The manufacturing process of MCFC was divided into four steps: molding the main body, presetting weak zones, splicing parts, and attaching additional layers. The process is shown in Figure 12, and the implementation process is as follows.

(1) Molding the main body: Under room temperature (25 °C), the mold was cleaned up, and the low-temperature grease was daubed on the mold surface. Polyester plastic film was cut and pasted on the mold according to the mold shape. The fabric layers with the ply sequences were laid. The adhesive solution with epoxy resin, benzene dimethylamine, and butyl phthalate was made and stirred evenly in accordance with a weight proportion of 10:2:1. Seal the mold, cover it with a vacuum bag, and turn on the vacuum pump to vacuum for 5 h.
(2) Presetting weak zones: The cover was de-molded, and the burrs were removed. Drill holes in the cover frame according to the assembly requirements. The cover was divided at the position of the weak zone according to the design purpose using mechanical cutting.
(3) Splicing parts: Assemble the retaining frame and the sub-covers together with the prime seam according to its original position, and fix the positions with adhesive solution (epoxy-resin system) for 30 min (expediting setting). Inject the adhesive solution into the triangle groove and keep it at room temperature for 12 h.
(4) Attaching additional layers: After curing, the weak zone surface was burnished and kept smooth. The additional layers on both sides were pasted with an epoxy-resin adhesive solution in accordance with the size requirements and then kept at 50 °C for 4 h.

### 4.2. Manufacturing Process

**Figure 13:**
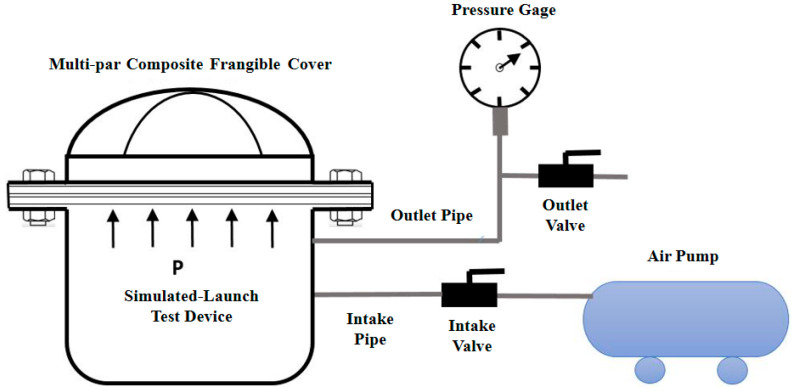
Simulated-launch test device.

**Figure 14:**
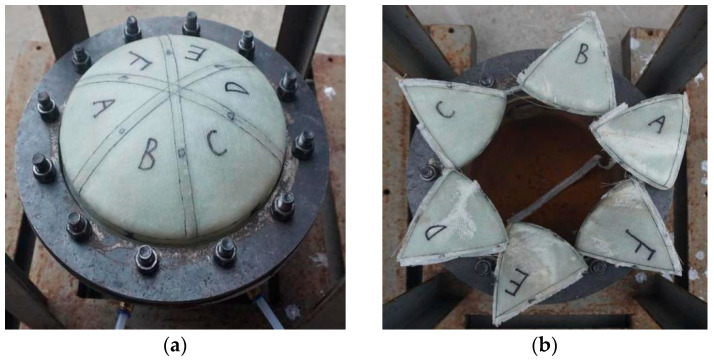
Experimental process of the multi-part composite frangible cover: (**a**) Before destructive test; (**b**) Ultimate failure.

Based on the dimensions of the MCFC, a simulated-launch test device was developed to emulate the gas flow imposed on the cover, as shown in Figure 13. The flange of the launch canister and the retaining frame of the cover were attached with a steel clamp ring, two rubber gaskets, and twelve bolts. The pressure loads on the MCFC could be applied with the gas pump according to the test conditions. Pressure data in the launch canister could be monitored in real-time with the pressure gage. The experimental process is shown in Figure 14.

The failure test showed that the corner weak zone (Area 3) was damaged first. The failure extended to the adjacent weak zones with increasing pressure in the launch canister. Finally, the sub-covers flew out in six petals. The duration of the failure process was short enough to ensure the simultaneous destruction of all weak areas. This phenomenon was consistent with the simulation results.

The test failure pressure results are shown in Table 5. The experimental results show that the failure pressure is repeatable, which validates the reliability of the design and manufacture project in this paper. The experimental failure pressure is close to the simulation value, with a mean error of 7.9%, indicating the veracity of the numerical simulation. The differences occurred between calculation results and test data because manufacturing deviations and impurity defects existed in the cover, which could reduce the strength. Also, the simulation model was simplified compared with the actual situation.

## 5. Conclusions

A new MCFC was designed and fabricated in this paper. The failure behavior of MCFC was researched using numerical simulation and experimental methods. The main works and conclusions in this paper are listed as follows:

(1) The variation of the strength of weak zones with lap parameters was analyzed. The equal strength design criterion was proposed to design the lap parameters of weak zones in the MCFC.
(2) A finite element model was used to perform numerical simulations during the separation process of the MCFC. The results show that the MCFC took only 0.2 ms from the initial damage to the complete separation, which basically guaranteed that the weak zones were destroyed simultaneously.
(3) The manual lay-up process and VARI method were combined to form MCFC. A simulated-launch test device was developed to conduct the failure separation tests. The test results show that the MCFC had stable bursting performance and that the sub-covers could be scattered along the preset trajectory. The average error of failure pressure between the test result and the simulation value was 7.9%, verifying the designed MCFC’s feasibility and rationality.

## Figures and Tables

**Table 1:** Parameters of the weak zones.

Weak Zone Type	Area	*L*/mm	*t*/mm
Spherical	Area 1	4	0.1
	Area 2	6	0.2
	Area 3	6	0.1
Cylindrical	Area 4	4	0.2

**Table 2:** Ply thickness of the multi-part composite frangible cover.

Area	Spherical Surface	Vertical Surface	Retaining Frame
Thickness/mm	1.8	2.4	2.4
Ply numbers	12	16	16

**Table 3:** Optimized ply angles and sequences of the multi-part composite frangible cover.

Position in the Cover	Ply Angles and Sequences
Spherical surface	[0_3_/30/0/30]_s_
Vertical surface	[−30_2_/30/45/0_3_/45]_s_
Frame	[−30_2_/30/45/0_3_/45]_s_

**Table 4:** Mechanical properties of the main body.

*E*_1_/GPa	*E*_2_/GPa	*μ*_12_	*G*_12_/GPa	*X_t_*/MPa	*X_c_*/MPa	*Y_t_*/MPa	*Y_c_*/MPa	*S*/MPa
18.89	18.89	0.11	3.31	344.91	261.52	344.91	261.52	58.82

**Table 5:** Failure pressure results of multi-part composite frangible cover.

Test Number	Simulation Pressure/MPa	Test Pressure/MPa	Errors/%
1#	0.78	0.728	7.7
2#		0.713	8.6
3#		0.722	7.4

## Data Availability

The data used to support the findings of this study are included within the article and available from the corresponding author upon request.
